# Expression of the Gene for Resistance to Phaseolotoxin (*argK*) Depends on the Activity of Genes *phtABC* in *Pseudomonas syringae* pv. phaseolicola

**DOI:** 10.1371/journal.pone.0046815

**Published:** 2012-10-08

**Authors:** Selene Aguilera, Susana De la Torre-Zavala, José Luis Hernández-Flores, Jesús Murillo, Jaime Bravo, Ariel Alvarez-Morales

**Affiliations:** 1 Departamento de Ingeniería Genética, Centro de Investigación y de Estudios Avanzados (CINVESTAV), Irapuato, Guanajuato, México; 2 Instituto de Biotecnología, Facultad de Ciencias Biológicas, Universidad Autónoma de Nuevo León, Nuevo León, México; 3 Departamento de Producción Agraria, Universidad Pública de Navarra, Pamplona, Spain; University of Helsinki, Finland

## Abstract

The bacterium *Pseudomonas syringae* pv. phaseolicola produces phaseolotoxin in a temperature dependent manner, being optimally produced between 18°C and 20°C, while no detectable amounts are present above 28°C. Phaseolotoxin is an effective inhibitor of ornithine carbamoyltransferase (OCTase) activity from plant, mammalian and bacterial sources and causes a phenotypic requirement for arginine. To protect the cell from its own toxin, *P. syringae* pv. phaseolicola synthesizes a phaseolotoxin-resistant OCTase (ROCT). The ROCT is the product of the *argK* gene and is synthesized only under conditions leading to phaseolotoxin synthesis. The *argK* gene is included in a chromosomal fragment named Pht cluster, which contains genes involved in the synthesis of phaseolotoxin. The aim of the present work was to investigate the possible involvement of other genes included in the Pht cluster in the regulation of gene *argK*. We conducted transcriptional analyses of *argK* in several mutants unable to produce phaseolotoxin, transcriptional fusions and electrophoretic mobility shift assays, which allowed us to determine that genes *phtABC*, located within the Pht cluster, participate in the transcriptional repression of gene *argK* at temperatures not permissive for phaseolotoxin biosynthesis. This repression is mediated by a protein present in both toxigenic and nontoxigenic strains of *P. syringae* and in *E. coli*, and requires the coordinated participation of *phtA*, *phtB* and *phtC* products in order to carry out an efficient *argK* repression.

## Introduction

Production of phaseolotoxin, a non host specific toxin, has been described in *Pseudomonas syringae* pv. phaseolicola, which infects bean (*Phaseolus vulgaris* L), *P. syringae* pv. actinidiae, which infects kiwi (*Actinidia chinensis*), and in strain CFBP3388 of *P. syringae* pv. syringae, isolated from vetch (*Vicia sativa*) [1], [2], [3]. There are natural strains of these pathovars that do not produce the toxin and that do not contain the DNA responsible for its synthesis [3], [4], [5], [6] indicating that the ability to produce phaseolotoxin has been acquired after pathovar delineation as a recent event [7]. Analysis of the conservation of the phaseolotoxin biosynthesis genes in a broad collection of *P. syringae* pv. phaseolicola and pv. actinidiae strains, as well as in pv. syringae strain CFBP3388, suggests that genes for the biosynthesis of phaseolotoxin have a complex evolutionary history and have been acquired by pathovars of *P. syringae* at least twice during evolution [6].

The production of phaseolotoxin is temperature dependent, being optimally produced between 18°C and 20°C, while no detectable amounts are present above 28°C [8], [9], [10]. Phaseolotoxin is composed of two moieties: the inorganic moiety, N^δ^ N′-sulfodiaminophosphinyl, and the L-ornithyl-alanyl-homoarginine tripeptide [1], [11]. Targets of this toxin are the enzymes ornithine carbamoyltransferase (OCTase; EC 2.1.3.3) [12], which catalyzes the formation of citrulline from ornithine and carbamoylphosphate in the arginine biosynthetic pathway, and ornithine decarboxylase, which participates in the biosynthesis of polyamines [13]. Phaseolotoxin is an effective inhibitor of OCTase activity from plant, mammalian and bacterial sources and causes a phenotypic requirement for arginine. This property led to the development of a rapid bioassay that evaluates growth inhibition of a bacterial culture exposed to this toxin [14].

To protect itself from its own toxin, *P. syringae* pv. phaseolicola synthesizes a phaseolotoxin-resistant OCTase (ROCT) [12], [15], [16], [17], [18]. The ROCT, which is the product of the *argK* gene and is expressed under conditions leading to phaseolotoxin synthesis, is a polypeptide composed of 327 amino acid residues with a molecular mass of 36.52 kDa, [18], [19], [20]. ROCT is necessary for *P. syringae* pv. phaseolicola under conditions of phaseolotoxin synthesis, because it ensures an optimal supply of the arginine required for growth and synthesis of phaseolotoxin [21].

Several efforts have been made to determine how *argK* is regulated. It has been postulated that *argK* might be regulated at 28°C under negative control by a repressor protein [20]. This repressor protein could bind to specific DNA motifs found in the *argK* promoter that have been postulated to be involved in thermoregulation of phaseolotoxin synthesis [22], [23]. Additionally, carbamoylphosphate is able to induce *argK* expression, bypassing the temperature control [21]. Since carbamoylphosphate is a compound resembling the inorganic moiety of phaseolotoxin, N^δ^ N′-sulfodiaminophosphinyl, these results suggested that *argK* is being directly regulated by a molecule that may be a precursor of phaseolotoxin and only indirectly by temperature [21]. On the other hand, the OCTase present under conditions nonpermissive for phaseolotoxin synthesis in *P. syringae* pv. phaseolicola, encoded by gene *argF*, is negatively regulated by ArgR [24]. However, production of ROCT and synthesis of phaseolotoxin occur independently of ArgR. Therefore there is not any apparent metabolic link between the genes for phaseolotoxin synthesis/resistance and the genes involved in the primary metabolism [24].

**Figure 1:**
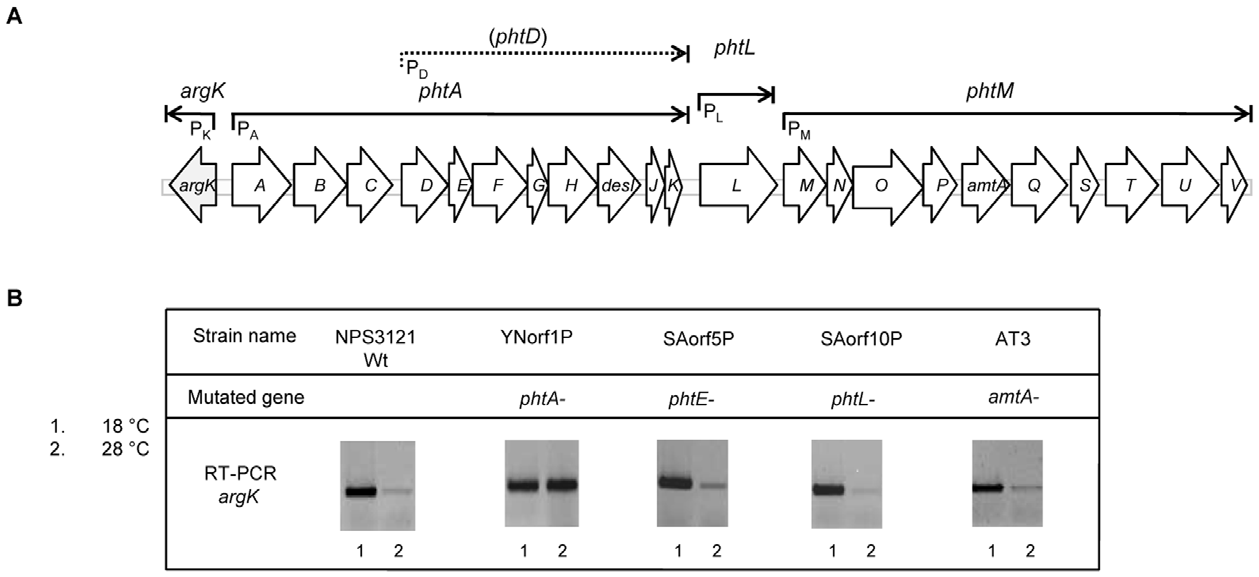
Participation of genes from the phaseolotoxin biosynthesis cluster in the expression of gene *argK* from *P. syringae* pv. phaseolicola NPS3121. A. Graphic representation of the phaseolotoxin biosynthesis cluster (Pht cluster) of *P. syringae* pv. phaseolicola NPS3121. The Pht cluster contains five transcriptional units, including two monocistronic (*argK* and *phtL*) and three polycistronic operons (*phtA*, *phtD* and *phtM*) [25]. There is a secondary promoter (P_D_) capable of driving the expression of genes from *phtD* to *phtK*. Genes are represented by arrows, with the direction of the arrow indicating the direction of transcription. B. Analysis of the *argK* transcriptional pattern in *P. syringae* pv. phaseolicola NPS3121 and polar mutants by reverse transcription-PCR. RT-PCR amplicons were separated by electrophoresis, and reversed images of the gels are shown; the strains analyzed and the corresponding mutated genes are described on top of their corresponding lanes. The small numbers under the lanes represent the temperature at which expression was assayed: 1 indicates 18°C and 2 indicates 28°C.

Genes required for the biosynthesis of and resistance to phaseolotoxin are grouped in the so-called Pht cluster and are included into a genomic region that has the characteristics of a pathogenicity island [15], [25], [26], [27], [28]. The Pht cluster is composed of 23 genes and it is flanked by insertion sequences and transposases. The 23 genes are organized in five transcriptional units (Figure 1A), two monocistronic and three polycistronic, with one of them overlapping a larger operon. Mutagenesis of 14 genes within the Pht cluster resulted in three mutants showing low levels of toxin production, while a Tox- phenotype was shown for the eleven remaining mutants [25].

The two-component system and global regulators GacS/GacA also participate in the regulation of phaseolotoxin biosynthesis [29], and in a *gacA* ^−^ background it was evident the downregulation of genes within operons *phtA*, *phtD*, *phtL* and *phtM*, whose expression was negligible at 18°C. Interestingly, gene *argK* losses its temperature-dependent expression in a *gacA* ^−^ background and, in contrast with the rest of the genes within the Pht cluster, it becomes constitutive at both 18°C and 28°C [29].

We investigated the possible involvement of other genes included in the Pht cluster in the regulation of the *argK* gene. To this end, we analyzed the transcription of *argK* in several mutants unable to produce phaseolotoxin, and we also conducted electrophoretic mobility shift assays, which allowed us to determine that genes *phtABC*, included in the Pht cluster, are required to control *argK* transcription in response to temperature in *P. syringae* pv. phaseolicola NPS3121. We also report that to carry out an efficient *argK* repression, it is necessary the coordinated participation of the products of *phtA*, *phtB* and *phtC*.

## Results

### *argK* transcriptional analysis in a group of mutants

We analyzed the effect on *argK* expression of mutations on different genes of the Pht cluster, including polar mutants YNorf1P, SAorf5P, SAorf10P and AT3, altered in genes *phtA*, *phtE*, *phtL* and *amtA*, respectively (Table 1) [25], [30]. To assess the expression pattern of gene *argK* at 18°C and 28°C in these mutants with respect to the wild type strain NPS3121, we conducted Reverse Transcription-PCR analysis (RT-PCR) aimed to amplify specific fragments derived from cDNA.

**Table 1:** Bacterial strains and plasmids.

Strain or plasmid	Relevant characteristics	Reference or source
**Bacterial strains**		
*Escherichia coli*		
DH5α	*supE44* Δ*lacU169* (Φ80*lacZ*Δ*M15*) *hsdR17 recA1 endA1 gyrA96 thi-1 relA1* Nal^r^	[41]
*P. syringae*		
pv. phaseolicola		
NPS3121	Wild type, Tox^+^	[27]
CYL233	Wild type, Tox^−^	[5]
YNorf1P	Tc^r^; *phtA*::*tet* polar mutant of NPS3121	[25]
SAorf5P	Tc^r^; *phtE*::*tet* polar mutant of NPS3121	[25]
SAorf10P	Km^r^; *phtL*::*uidA*-*aph* polar mutant of NPS3121	[25]
AT3	Km^r^; *amtA*::*aph* polar mutant of NPS3121	[30]
pv. tomato DC3000	Rif^r^ derivative of NCPPB1106	[42]
pv. syringae B728a	Wild type; Rif^r^, Cu^r^, Str^r^	[43]
pv. glycinea PG4180	Wild type	[35]
**Plasmids**		
pUCP20	*Pseudomonas-E. coli* shuttle vector; Ap^r^; 3.89-kb; *lac*Z'	[31]
pWM6	Source of GUS cassette	[47]
pSAK	Ap^r^; *argK* in pUCP20	This study
pSAK-A	Ap^r^; *argK-phtA* in pUCP20	This study
pSAK-B	Ap^r^; *argK-phtB* in pUCP20	This study
pSAK-C	Ap^r^; *argK-phtC* in pUCP20	This study
pSAK-AB	Ap^r^; *argK-phtAB* in pUCP20	This study
pSAK-BC	Ap^r^; *argK-phtBC* in pUCP20	This study
pSAK-AC	Ap^r^; *argK-phtAC* in pUCP20	This study
pSAK-ABC	Ap^r^; *argK-phtABC* in pUCP20	This study
pSAF	Ap^r^; *uidA* in pUCP20	This study
pSAFP_KA_	Ap^r^; *uidA*-P_KA_ in pUCP20	This study
pSAF-A	Ap^r^; *uidA*-P_K_-*phtA* in pUCP20	This study
pSAF-B	Ap^r^; *uidA*-P_K_-*phtB* in pUCP20	This study
pSAF-C	Ap^r^; *uidA*-P_K_-*phtC* in pUCP20	This study
pSAF-AB	Ap^r^; *uidA*-P_K_-*phtAB* in pUCP20	This study
pSAF-BC	Ap^r^; *uidA*-P_K_-*phtBC* in pUCP20	This study
pSAF-AC	Ap^r^; *uidA*-P_K_-*phtAC* in pUCP20	This study
pSAF-ABC	Ap^r^; *uidA*-P_K_-*phtABC* in pUCP20	This study

In mutant YNorf1P, the *argK* gene showed an increased expression at 28°C, unlike what happens in strain NPS3121 at the same temperature (Figure 1B), indicating that a mutation on the *phtA* operon resulted in alleviation of the repression of *argK* at a nonpermissive temperature for phaseolotoxin synthesis. These results are compatible with previous reports postulating that in *P. syringae* pv. phaseolicola, the *argK* gene could be regulated under negative control by a repressor protein at 28°C [20]. Conversely, transcription of gene *argK* in mutants SAorf5P, SAorf10P and AT3, affected in genes *phtE*, *phtL* and *amtA*, respectively, was similar to that in wild type strain NPS3121 at both temperatures. Since a mutation into gene *phtE*, which belongs to the *phtA* operon, did not modify *argK* expression at 28°C, it is likely that only genes located upstream to *phtE* could be participating in *argK* regulation (Figures 1A;1B).

### Mutant YNorf1P showed an increase in OCTase activity at 28°C

**Figure 2:**
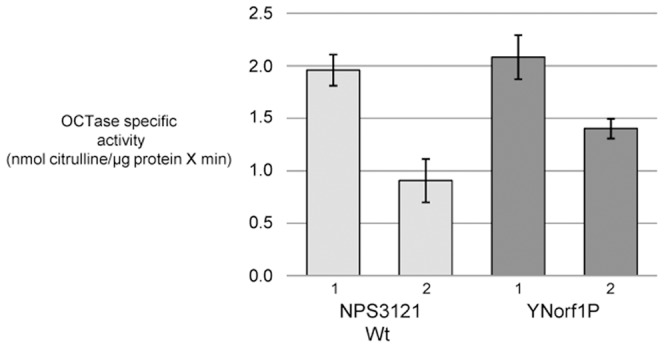
Ornithine carbamoyltransferase (OCTase) specific activity of strains *P. syringae* pv. phaseolicola NPS3121 and YNorf1P. The strains analyzed are described under their corresponding value bars; YNorf1P is a derivative of NPS3121 with *phtA* inactivated by site-directed mutagenesis. The small numbers under the bars represents the temperature at which expression was carried out: 1 indicates 18°C and 2 indicates 28°C. Error bars represent standard deviation from triplicate samples.

Based in the previous RT-PCR analyses showing an increase in *argK* expression at 28°C in mutant YNorf1P (Figure 1B) and considering that the *argK* gene codes for the phaseolotoxin-resistant OCTase, we analyzed the OCTase specific activity at 28°C in YNorf1P in comparison to that of the wild type strain NPS3121. In order to discard any activity corresponding to the phaseolotoxin-sensitive OCTase, we preincubated the reaction mixture with a phaseolotoxin containing supernatant; later, OCTase activity was determined as previously reported [24]. The results obtained are shown in Figure 2. In agreement with the results obtained by RT-PCR, we observed a significant increase in the OCTase activity in YNorf1P compared with NPS3121, indicating that the *argK* expression level observed at 28°C was directly related to an increase in the OCTase activity.

### Effect of *phtABC* genes on *argK* expression pattern in *P. syringae* pv. phaseolicola strain CYL233

Since *argK* transcription was unambiguously de-repressed at 28°C in mutant YNorf1P, we decided to determine which genes from the *phtA* operon could participate in *argK* regulation. The *phtA* operon contains 11 genes, from *phtA* to *phtK*, which are transcribed divergently to the *argK* gene, although it also possesses an internal promoter immediately downstream of *phtC*, capable of driving expression of *phtD* to *phtK* (Figure 1A). Additionally, RT-PCR analyses indicated that a polar mutation in gene *phtE* did not modify *argK* expression at 18°C and 28°C (Figure 1B). Therefore, we focused our analysis in the participation of genes *phtA*, *phtB* and *phtC* in the *argK* regulation considering that *phtE* gene belongs to the *phtD* operon. To carry out our experiments we decided to use the *P. syringae* pv. phaseolicola wild type strain CYL233 with the aim to discard the participation of other genes from the Pht cluster. It was reported that strain CYL233 is naturally unable to synthesize phaseolotoxin because it lacks the entire Pht cluster for phaseolotoxin biosynthesis [5], [6]. PCR analyses using primers directed to all genes from the Pht cluster did not yield any amplification product using DNA from CYL233 as template (data no shown), supporting the idea that this strain really lacks the entire Pht cluster.

**Figure 3:**
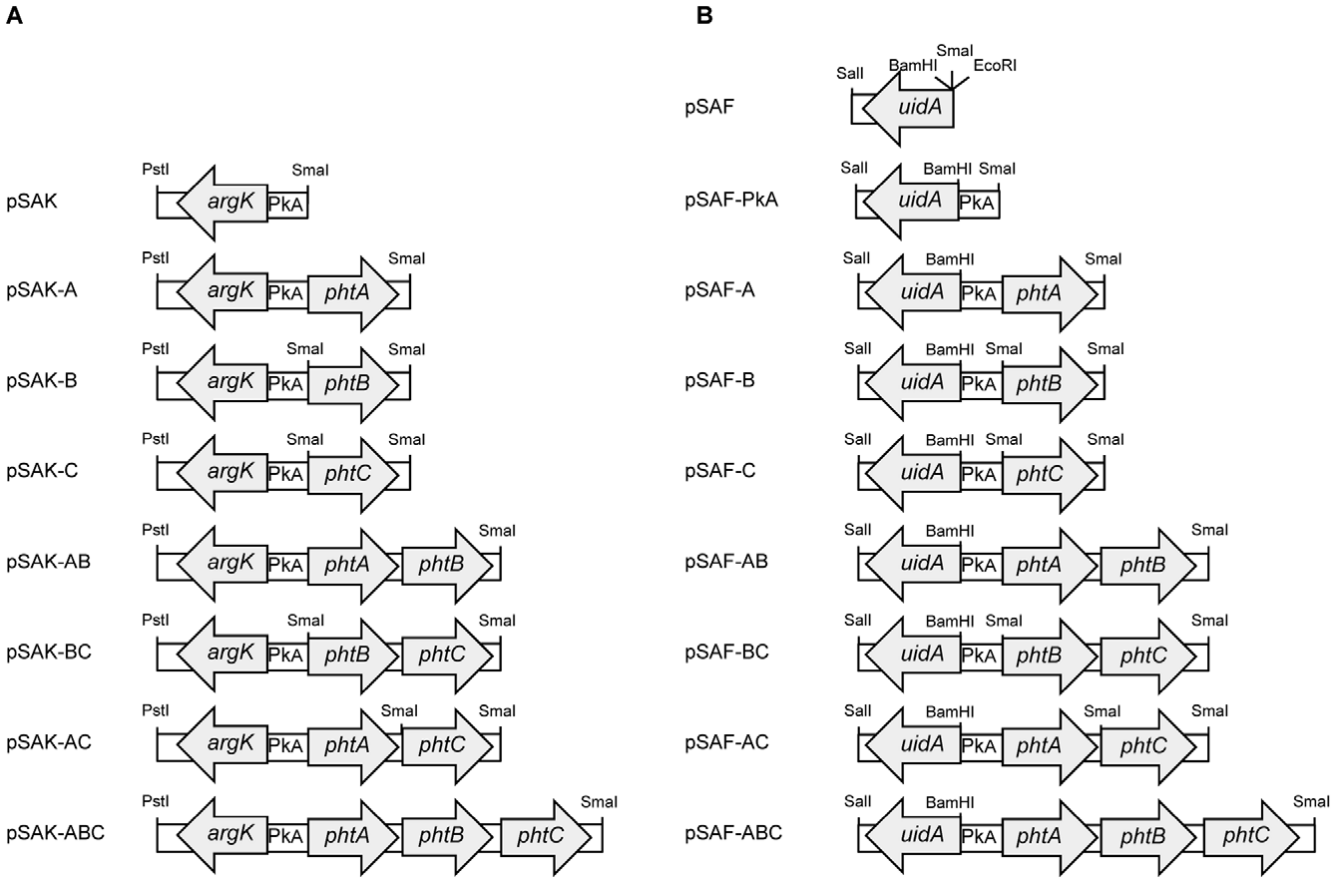
Schematic representation of plasmid clones used for expression analyses. Each arrow represents each gene amplified and cloned into pUCP20. Each plasmid name is indicated at left. The direction of the arrow indicates the direction of transcription. All restriction sites for PstI, SmaI, SalI, BamHI and EcoRI are shown. A. Constructs used in Northern blot analyses. B. Constructs to evaluate the activity of the *argK* gene using transcriptional *uidA* fusions.

Plasmids pSAK; pSAK-A; pSAK-B; pSAK-C; pSAK-AB; pSAK-BC; pSAK-AC and pSAK-ABC were constructed containing genes *argK*; *argK*-*phtA*; *argK*-*phtB*; *argK*-*phtC*; *argK*-*phtAB*; *argK*-*phtBC*; *argK*-*phtAC* and *argK*-*phtABC* cloned into pUCP20, respectively (Table 1; Figure 3A). These constructions were electroporated into *P. syringae* pv. phaseolicola strain CYL233 and the *argK* expression pattern was evaluated by Northern blot analysis.

#### a) *argK* expression in *P. syringae* pv. phaseolicola CYL233 wild type strain

**Figure 4:**
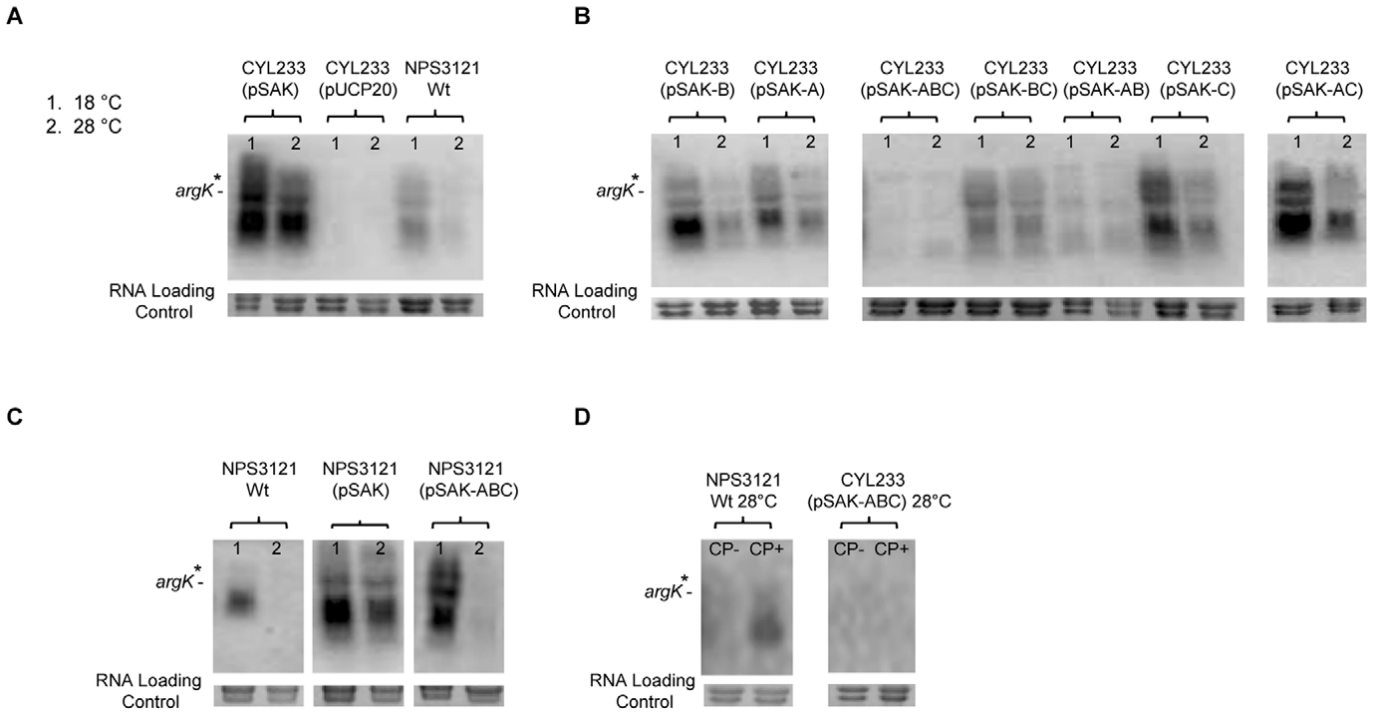
Effect of cloned *argK* and *phtABC* genes on the *argK* expression in *P. syringae* pv. phaseolicola. The expression of *argK* was evaluated by Northern blot in derivatives of strain CYL233 (nontoxigenic; panels A and B) and strain NPS3121 (toxigenic, panel C) harboring plasmids with diverse combinations of genes *argK*, *phtA*, *phtB* and *phtC*, as indicated above each blot (see Figure 3 for a description of each plasmid). Panel D shows the expression of *argK* in the absence or presence of carbamoylphosphate (CP) in strains NPS3121 and CYL233 at 28°C. Blots were hybridized with an internal probe specific for *argK*, and the signal corresponding to the monocistronic *argK* RNA is marked. The asterisks indicate the position of a band of approximately 2.3-kb, corresponding to a previously described possible alternative *argK* transcript. Strain CYL233(pUCP20) was used as negative control of *argK* expression, whereas the wild type strain NPS3121 was used as a positive control. The numbers on top of the Northern blots represent the temperatures at which expression was assayed: 1 indicates 18°C and 2 indicates 28°C.

Strains were grown in M9 medium at 18°C and 28°C. In strain CYL233(pSAK) we observed *argK* transcripts at both temperatures, as it occurs in mutant YNorf1P and in contrast with the expression observed in the wild type strain NPS3121, where *argK* expression is only observed at 18°C (Figure 4A). These results indicate that the molecule that repress *argK* gene expression at 28°C is either not present or inactive in the wild type strain CYL233, suggesting that this repressor molecule could be coded within the Pht cluster. On the other hand, the *argK* expression observed at 18°C in strain CYL233 was similar to that in strain NPS3121 (Figure 4A), showing that the Pht cluster is dispensable to carry out an efficient *argK* transcription.

#### b) Effect of *phtA*, *phtB* and/or *phtC* on *argK* transcription in strain *P. syringae* pv. phaseolicola CYL233

We evaluated the effect of *phtA*, *phtB* and *phtC* on *argK* transcription. To achieve this we constructed different plasmids containing *argK* and a combination of genes *phtA*, *phtB* and *phtC* transcribed divergently to *argK* (Figure 3A) that were transferred to strain *P. syringae* pv. phaseolicola CYL233. Northern blot analyses revealed a clear *argK* transcriptional repression effect when this gene was transcribed together with *phtABC* in strain CYL233 at both temperatures (Figure 4B). This repression effect was not so efficient with other combination of genes *phtA*, *phtB* and *phtC* (Figure 4B).

### Effect of *phtABC* genes on *argK* transcription *in trans* in strain *P. syringae* pv. phaseolicola NPS3121

To evaluate this, we introduced plasmids pSAK and pSAK-ABC in the wild type strain NPS3121 and evaluated the expression pattern of *argK* in each derivative (Figure 4C). In strain NPS3121(pSAK), we could still observe *argK* expression at 28°C (Figure 4C). This result was not unexpected, since pSAK is a plasmid that occurs in multiple copies [31] and the cloned *argK* gene would probably titrate the putative repressor. However, in a NPS3121 derivative containing pSAK plus genes *phtABC* (plasmid pSAK-ABC, Figure 3), the transcription of *argK* was regulated by temperature.

### Induction of *argK* expression with carbamoylphosphate in strain *P. syringae* pv. phaseolicola CYL233

A model of phaseolotoxin regulation has been proposed in which, at permissive temperature for phaseolotoxin production, 18°C, an inducer molecule could bind the postulated repressor molecule of *argK* to release it from the *argK* operator allowing its expression [21]. It has also been proposed that such inducer could be a precursor of phaseolotoxin. Carbamoylphosphate, which presents a similar structure to that of the inorganic moiety of phaseolotoxin, induces *argK* expression at 28°C [21]. We decided to determine whether this molecule was able to eliminate the *argK* transcriptional repression caused by *phtABC*. Northern blot analyses of RNA extracted from cells grown in M9 medium at 28°C until the end of the logarithmic phase, showed that there was not a de-repression effect caused by carbamoylphosphate in strain CYL233(pSAK-ABC) (Figure 4D).

### Transcriptional fusions to the *uidA* reporter gene in strain *P. syringae* pv. phaseolicola CYL233

In order to investigate the regulatory mechanism exerted by *phtA*, *phtB* and *phtC* products on *argK* transcription, we decided to construct transcriptional fusions to *uidA* (GUS) reporter gene (Figure 3B). Transcription of the GUS reporter gene was determined in the wild type strain CYL233 by a qualitative colorimetric assay using X-Gluc as substrate. The strains were grown in M9 medium at 18°C and 28°C, and the presence or absence of blue color in the culture medium was evaluated. Reporter activation was detected for cultures of CYL233(pSAF-P_KA_), indicating that the *argK* promoter (P_KA_) was able to drive *uidA* expression (Figure S1). Likewise, we observed *argK* promoter activity for all the constructions containing genes *phtA*, *phtB* or *phtC*, either by themselves or in combination (Figure S1). The only exception was CYL233(pSAF-ABC), containing genes *phtABC*, which resulted in cultures that did not develop any color; moreover, with this strain we could not detect even the background blue color produced with the promoterless construct, pSAF. These results complement our observations obtained by Northern blot hybridization, and support the hypothesis that the *phtABC* products participate in the transcriptional regulation of gene *argK*, interacting either directly or indirectly with the *argK* promoter.

### DNA electrophoretic mobility shift assay

Results obtained using transcriptional fusions suggest that the *phtA*, *phtB* and *phtC* products participate in the regulation of gene *argK* at the transcriptional level. To analyze the possibility that the *phtABC* products could bind to the promoter region of *argK* we performed an electrophoretic mobility shift assay. The *argK* promoter has been previously defined and shown to be a Pribnow (σ70) type promoter with appropriate −10 and −35 regions [19], [20].

**Figure 5:**
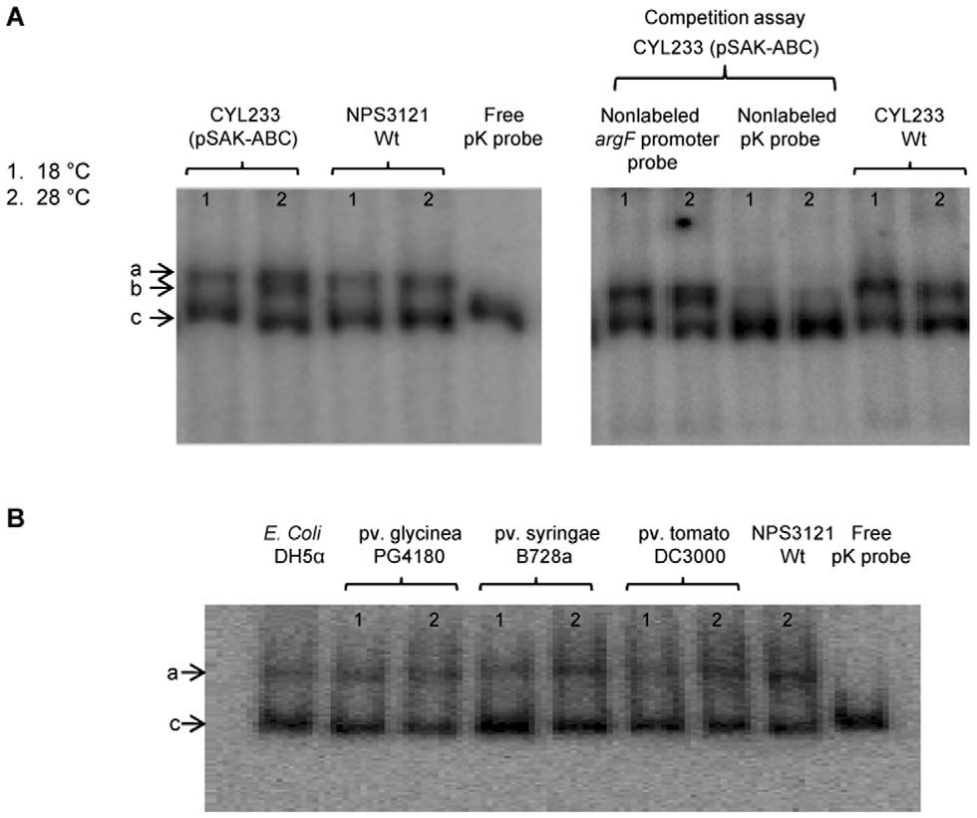
Gel retardation assay using an *argK* promoter (P_K_) probe. The strains analyzed are described on top of their corresponding lanes. A. Gel retardation and competition assays using *P. syringae* pv. phaseolicola NPS3121 and CYL233. B. Gel retardation assay using *E. coli* DH5α and *P. syringae* pv. glycinea PG4180, pv. syringae B728a and pv. tomato DC3000. The P_K_ probe plus extract from the wild type strain NPS3121 grown at 28°C was used as positive control. The extract from *E. coli* DH5α was obtained from cells grown in LB at 37°C. “a" and “b" indicate the position of retardation signals; “c" indicates the position of free probe. The numbers on top of each lane represent the temperatures at which the cells were grown: 1 indicates 18°C and 2 indicates 28°C.

Experiments were carried out by using a 288-bp DNA probe from the *argK* promoter region containing the −10 and −35 regions. A clear retardation signal of the P_K_ probe was observed when crude extracts from NPS3121, CYL233 and CYL233(pSAK-ABC) strains, grown at 18°C or 28°C in M9 medium, were added to the retardation mixture (Figure 5A). These results indicate that a molecule coded within the chromosome of *P. syringae* pv. phaseolicola was bound to this probe. Also, a second retardation signal was observed when crude extracts from CYL233(pSAK-ABC) strain grown at 28°C were added to the retardation mixture (Figure 5A). Specific binding to the *argK* promoter probe was demonstrated when the nonlabeled P_K_ probe efficiently replaced the labeled probe, causing the almost complete disappearance of the retardation signal. A nonlabeled *argF* promoter probe failed to compete the binding of the labeled P_K_ probe (Figure 5A); indicating that unspecific binding to the probe was not occurring.

We observed a second retardation signal when the P_K_ probe was incubated with crude extracts from strain CYL233 containing genes *phtABC* (Figure 5A); however, it is unlikely that the products of these genes will directly bind to the *argK* promoter. The deduced product of *phtA* belongs to the “P-loop containing nucleoside triphosphate hydrolases" superfamily (InterPro SSF52540), whereas there were no hits for the products of *phtB* and *phtC* in an InterProScan comparison. Therefore, these products probably do not have DNA binding domains that would suggest a possible function as repressor proteins. Additionally, database comparisons using available BLAST [32] did not find any similarity with proteins related to transcription factors or DNA binding proteins.

We decided to investigate whether the proteins binding the *argK* promoter were present in other *P. syringae* pathovars and in *E. coli*. Results are shown in Figure 5B, where we observed only one retardation signal in all strains, indicating that all analyzed strains produce the unknown protein that binds to the *argK* promoter.

## Discussion

Regulation of the phaseolotoxin biosynthesis cluster is very complex and there are at least three regulatory circuits that differentially affect its five transcriptional units. The global regulators GacA/GacS are required for the expression of all the transcriptional units, except for *argK*, which becomes constitutive in a *gacA^−^* mutant background [29]. Additionally, the expression of the *phtM* transcriptional unit depends on the activity of gene *phtL*, also included in the Pht cluster [25]. Finally, the global regulator IHF (Integration Host Factor) participates in the repression control of the *phtD* operon [33].

Expression of gene *argK*, conferring resistance to phaseolotoxin, appears to depend on different regulatory circuits than the rest of the Pht cluster. It is well known that fragments containing genes involved in toxin synthesis commonly carry regulatory elements that directly control the expression of biosynthetic genes [34]. An example are *corP*, *corS*, and *corR* genes, a modified two-component regulatory systems involved in the regulation of coronatine synthesis by *P. syringae* pv. glycinea PG4180. These regulatory genes have been located within the coronatine gene cluster [34], [35], [36]. In support of early predictions about *argK* regulation [20], [21], [22], [23], our results indicate that regulation of *argK* in *P. syringae* pv. phaseolicola NPS3121 is carried out by a repressor protein that prevents its expression at 28°C; additionally, we show that genes *phtABC*, from the Pht cluster, are essential for this repression.

The unknown protein participating in the repression of gene *argK* appears to be normally present in a variety of bacteria, because crude cell extracts from different *P. syringae* and *E. coli* strains contained a protein able to bind the *argK* promoter. This is not surprising, because it is well known that several regulatory genes involved in phytotoxin synthesis could also be coded outside the toxin biosynthesis cluster [34], [37], [38]. An example are the syringomycin and syringopeptin biosynthesis clusters from *P. syringae* pv. syringae, which are regulated by a two components regulatory system, GacS/GacA, and the regulator SalA, all coded outside the toxins gene clusters [37], [39]. Additionally, several genes located outside the Pht cluster are involved in phaseolotoxin synthesis and regulation in *P. syringae* pv. phaseolicola NPS3121. These genes include locus PSPPH_4550, coding for a putative nonribosomal peptide synthetase involved in phaseolotoxin production [29]; the GacS/GacA system, which is involved in the global regulation of phaseolotoxin biosynthesis genes [29] and the integration host factor, which participates in the repression of the *phtD* operon [33]. These facts, along with the results from this work support the idea that, following its acquisition by horizontal transfer, the Pht cluster has integrated into the regulatory circuits of *P. syringae* pv. phaseolicola.

The putative *argK* repressor protein was also present in the nontoxigenic strain *P. syringae* pv. phaseolicola CYL233, as shown by a specific retardation of the Pk promoter in gel-shift assays. Unlike what happened with other bacteria, we observed a second retardation signal when using extracts from strain CYL233 containing *phtABC* and grown at 28°C, which necessitates of further experiments for clarification. Nevertheless, it is unlikely that the products of genes *phtABC* bind directly to the *argK* promoter to repress transcription, because they did not contain any conserved regulatory or DNA-binding domain in comparison with the InterPro databases. Gene *phtA* belongs to the P-loop containing nucleoside triphosphate (NTP) hydrolases superfamily (InterPro SSF52540), whose members can function as kinases with very different specificities as different kinds of motor proteins and as batteries to drive reactions through conformational change [40], suggesting an enzymatic role for the corresponding *phtA* product. We can therefore speculate that the products of the *phtABC* genes participate in the biosynthesis of a phaseolotoxin precursor that can also bind the *argK* repressor molecule, leading to active repression. Genes *phtABC* lead to a very efficient repression of *argK* transcription in the nontoxigenic strain CYL233, when all were cloned in a high-copy number vector; remarkably, this repression was not alleviated at 18°C, a temperature at which *argK* is normally expressed. However, *argK* displayed the wild type expression pattern (i.e., expressed at 18°C but not at 28°C) when using the same construction in the toxigenic strain NPS3121. Carbamoylphosphate is one of the substrates for OCTase in the arginine biosynthesis pathway, and exogenously supplied carbamoylphosphate was shown to induce *argK* expression at 28°C in strain NPS3121 [21]. However, carbamoylphosphate did not relieve the repression of *argK* in strain CYL233. We do not have yet a satisfactory explanation for this result, although a likely possibility is that other genes of the Pht cluster are involved in the transformation of carbamoylphosphate into an inducer of *argK* transcription. This is the first study that identify genes involved in *argK* regulation in *P. syringae* pv. phaseolicola. Further work to understand this intriguing and interesting regulatory mechanism is currently under way in our laboratory.

## Materials and Methods

### Media, bacterial strains and supplements

The bacterial strains and plasmids used in this study are listed in Table 1. *Escherichia coli* strains were grown in Luria Bertani (LB) medium at 37°C [41]. *P. syringae* pathovars [42], [43] and mutant derivatives [25] were routinely grown on King's B medium [44] or M9 medium [41] at 18°C or 28°C. *P. syringae* pv. phaseolicola NPS3121 and *P. syringae* pv. phaseolicola CYL233 are referred to as wild type strains. Supplements were added to the following final concentrations: carbenicillin, 100 µg/ml; kanamycin, 50 µg/ml; tetracycline, 10 µg/ml and X-Gluc (5-bromo-4-chloro-3-indolyl-β-D-glucuronic acid), 20 µg/ml.

### Molecular biology techniques

Routine techniques were performed as described [41]. Purification of DNA from agarose gels and plasmid minipreps were performed using Qiagen columns and kits (Valencia, Ca, USA). Chromosomal DNA from *P. syringae* pv. phaseolicola was obtained as described [45]. DNA fragments used as probes for Northern blots were labelled with [α-^32^P]dCTP using the Rediprime random primer labeling kit (Amersham). Protein concentration in bacterial lysates was estimated by the procedure of Bradford [46].

### Analysis of *argK* transcriptional pattern by Reverse Transcription-PCR analysis

DNA-free RNA was obtained from cultures grown in M9 medium at 18°C and 28°C when they reached an O.D._600_ of 0.8. The RNA integrity was verified in a denaturing agarose gel and used for reverse transcription (RT) and PCR using the SuperScript One-Step kit (Invitrogen). Samples of RNA (20 ng) were used in each RT-PCR reaction. A 608-bp DNA amplicon from gene *argK* was obtained by RT-PCR using specific primers (Table S1). Controls used for the set of primers were: 1) PCR without the reverse transcription step to verify the absence of DNA; 2) RT-PCR performed without RNA templates to detect any contaminating DNA/RNA; 3) PCR performed using chromosomal DNA as template to ensure primer fidelity; 4) Amplification of a portion of the 23S ribosomal RNA operon using suitable primers as an internal control of the reaction. The RT reaction was performed at 50°C for 40 min, followed by PCR amplification at 94°C for 2 min for 1 cycle; 94°C for 35 s, 58°C for 30 s, 72°C for 1 min for 25 cycles; 72°C for 15 min for 1 cycle. The quantity of amplicon between samples was compared.

### OCTase activity assays

The OCTase activity was determined by measuring OCTase specific activity as previously described [24] by using 5 ml cultures of strains NPS3121 (wild type) and Ynorf1P (*phtA^−^*) grown in M9 medium at 28°C during 24 h. Cells were disrupted with a VirTis sonicator (model VirSonic 60), and 3 µl of this crude extract were used for the assay. The reaction mixtures were incubated at 37°C for 20 min and OCTase activity was determined. To eliminate OCTase activity from samples due to the phaseolotoxin-sensitive OCTase, coded by gene *argF*, phaseolotoxin-containing supernatant was added to the reaction mixture. To determine the amount of supernatant enough to achieve an efficient OCTase inhibition, we constructed a standard curve (OCTase activity vs. supernatant) (data no shown).The addition of a phaseolotoxin-containing supernatant to the reaction mixture and a preincubation for 25 min was made before OCTase activity was determined. OCTase specific activity was reported as nmol of citrulline produced per µg of protein per min at 37°C.

### Construction of plasmids containing *argK*, *phtA*, *phtB* and/or *phtC* genes

The *argK*, *phtA*, *phtB* and *phtC* genes were obtained by PCR using primers designed to include suitable restriction sites (Table S1). The *argK*, *argK-phtA*, *argK-phtAB* and *argK-phtABC* amplicons were cloned into the PstI-SmaI sites of the pUCP20 vector to create plasmids pSAK, pSAK-A, pSAK-AB, and pSAK-ABC, respectively. The *phtB*, *phtC* and *phtBC* amplicons were cloned into the SmaI site of pSAK to create pSAK-B, pSAK-C and pSAK-BC, respectively. The *phtC* amplicon was cloned into the SmaI site of pSAK-A to create pSAK-AC (Table 1, Figure 3A). All constructions were confirmed by restriction pattern and electroporated into *P. syringae* pv. phaseolicola.

### RNA extraction and Northern blot analysis

Total RNA was isolated from cultures of *P. syringae* pv. phaseolicola grown in M9 medium at 18°C or 28°C until an O.D._600_ of 0.8 using the TRIzol Reagent as recommended by the manufacturer (Invitrogen). Genomic DNA was removed by digestion with Rnase-free DNase (Roche). Samples of total RNA (25 µg) were denatured by treatment with formamide and separated by electrophoresis using 1.3% denaturing agarose gels. The RNA was transferred to Hybond N^+^ nylon membranes (Amersham) and cross-linked by exposure to UV radiation. Hybridization was performed using NorthernMax Prehybridization/Hybridization buffer (Ambion) and a DNA probe for the *argK* gene. The *argK* probe was labeled with [α-^32^P]dCTP by using the RediPrime Random Primer labeling kit. Hybridization was carried out overnight at 60°C. The membranes were washed twice with 2× SSC-0.1% sodium dodecyl sulfate (1× SSC is 0.15 M NaCl and 0.015 M sodium citrate) for 5 min at room temperature, followed by a wash with 1× SSC-0.1% sodium dodecyl sulfate for 15 min at 60°C. The membranes were exposed to a phosphorus screen and the signal detected in a Storm 860 apparatus. The analysis was made using ImageQuant version 1.1 (BioRad).

### Induction assays with carbamoylphosphate

Induction assays were conducted as previously reported [21] using cultures of *P. syringae* pv. phaseolicola grown in M9 medium at 28°C to an O.D._600_ of 0.8. Carbamoylphosphate disodium salt (Sigma) was added to the cultures at 1 mM and, after a 30 min incubation, RNA was purified from cultures.

### Colorimetric GUS assays of transcriptional *uidA* fusions

The 1.8-kb EcoRI-SalI fragment containing a promoterless *uidA* gene was obtained from pWM6 [47]. This fragment was cloned into pUCP20 to construct pSAF, containing the promoterless *uidA* gene preceded by a SD sequence. Fragments containing the *argK* promoter and *phtA* promoter (P_KA_), P_KA_-*phtA*, P_KA_-*phtAB*, P_KA_-*phtABC* were obtained by PCR using primers designed to include suitable restriction sites (Table S1) and were cloned into the BamHI-SmaI sites of pSAF to create pSAF-P_KA_, pSAF-A, pSAF-AB and pSAF-ABC respectively. The *phtB*, *phtC* and *phtBC* amplicons were cloned into the SmaI site of pSAF-P_KA_ to made pSAF-B; pSAF-C and pSAF-BC respectively. The *phtC* amplicon was cloned into the SmaI site of pSAF-A to create pSAF-AC (Table 1, Figure 3B). The orientations were determined by restriction pattern and constructions were electroporated into strain *P. syringae* pv. phaseolicola CYL233. The activation of the GUS reporter was determined by a colorimetric assay [48]. To analyze GUS activity, *P. syringae* pv. phaseolicola strains carrying *uidA* fusions were grown in M9 medium at 18°C and 28°C and when they reached an O.D._600_ of 0.8 were used. Culture dilutions (1∶10) were made and X-Gluc (20 µg/ml) was added. They were incubated at 18°C and 28°C during 6 h and 3 h, respectively, and the presence or absence of blue color in the culture medium was evaluated.

### DNA Electrophoretic mobility shift assay

DNA electrophoretic mobility shift was determined as previously reported [24], [49]. A 288-bp DNA probe from the *argK* promoter (P_K_) containing −10 and −35 regions, was obtained by PCR using specific primers (Table S1). The probe was labeled with [γ-^32^P]dATP by using the T4 polynucleotide kinase as recommended by the manufacturer (Invitrogen). Crude extract from cultures were obtained by sonication. The cell pellet was resuspended in extraction buffer [25 mM Tris (pH 8.0), 0.1 mM EDTA, 1 mM dithiothreitol, 10% glycerol, 0.02 mM phenyl-methyl-sulfonyl-fluoride] and sonicated using a VirTis sonicator (model VirSonic 60) with three pulses for 10 s with pauses for 10 s. Protein concentration in bacterial lysates was estimated by the procedure of Bradford [46]. The retardation reaction mixture [20 mM Tris (pH 8.0), 50 mM KCl, 1 mM EDTA, 1 mM dithiothreitol, 5% glycerol, 10 ng/µl of poly(dI-dC), 5 µg of crude extract], was preincubated for 15 min at room temperature, the radioactively labeled probe was added, and the mixture was incubated for 25 min at room temperature. Samples were loaded onto 5% polyacrylamide gels and separated at 7 mA for 90 min using Tris-acetate-EDTA buffer. In the competition assays, nonlabelled *argF* promoter probe and nonlabeled pK probe were added. Gels were dried and exposed to phosphorus screen and signal detected in a Storm 860 apparatus and analyzed using ImageQuant version 1.1 (BioRad).

## Supporting Information

Figure S1 **Qualitative assays of** ***argK*** **expression using transcriptional fusions with the** ***uidA*** **gene.** The wild type strain CYL233 was transformed with plasmids containing a transcriptional fusion between the promoter of *argK* and the *uidA* gene, and different combinations of genes *phtA*, *phtB* and/or *phtC* (see Figure 3 for a description of each plasmid). Cultures were then incubated in the presence of X-Gluc at 18°C and 28°C during 6 h and 3 h, respectively. Each strain is indicated above the corresponding tube. The activation of the GUS reporter was evaluated as presence or absence of blue color in the culture medium.
(TIF)

Table S1 **List of primers used for the amplification of** ***pht*** **genes.**
(DOC)
