# Supplementary figures and images for: Expression of the Gene for Resistance to Phaseolotoxin (argK) Depends on the Activity of Genes phtABC in Pseudomonas syringae pv. phaseolicola
Source: PLoS One. 2012 Oct 8;7(10):e46815. doi: 10.1371/journal.pone.0046815 (PMC3466206; doi:10.1371/journal.pone.0046815)

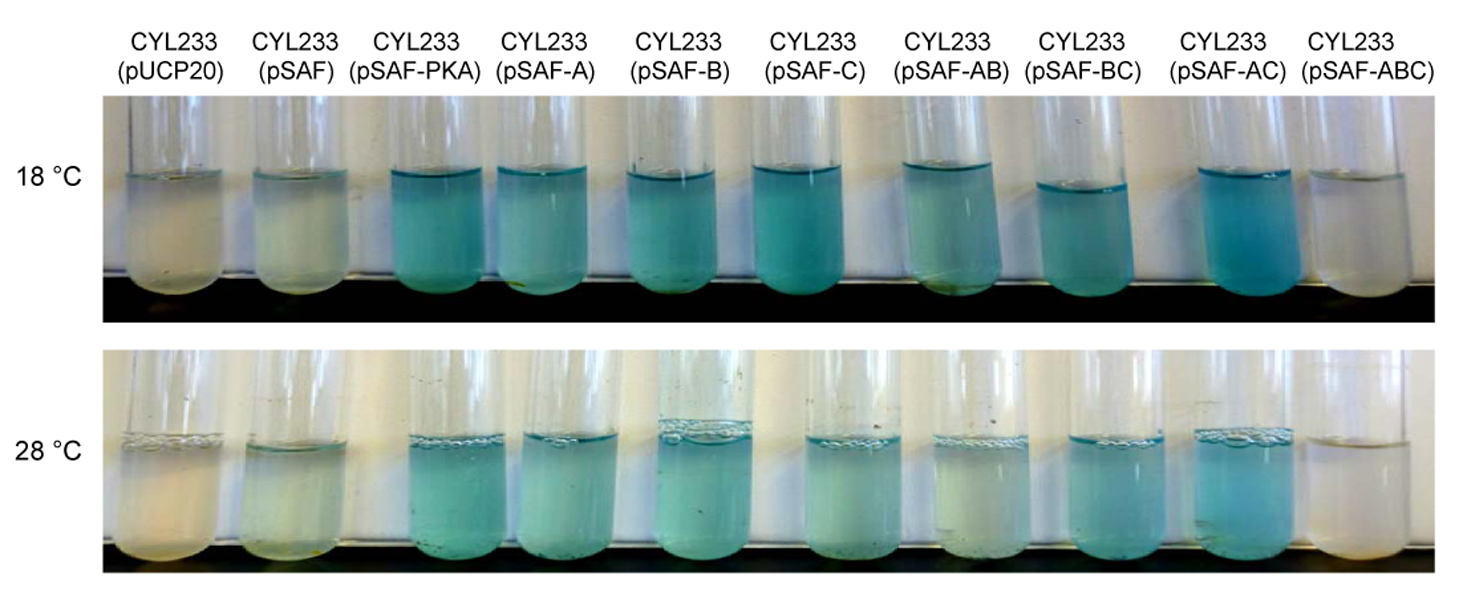

Supplement: Figure S1 — Qualitative assays of argK expression using transcriptional fusions with the uidA gene. The wild type strain CYL233 was transformed with plasmids containing a transcriptional fusion between the promoter of argK and the uidA gene, and different combinations of genes phtA, phtB and/or phtC (see Figure 3 for a description of each plasmid). Cultures were then incubated in the presence of X-Gluc at 18°C and 28°C during 6 h and 3 h, respectively. Each strain is indicated above the corresponding tube. The activation of the GUS reporter was evaluated as presence or absence of blue color in the culture medium. (TIF) [file pone.0046815.s001.tif]
