# Supplementary material for: Expression of the Gene for Resistance to Phaseolotoxin (argK) Depends on the Activity of Genes phtABC in Pseudomonas syringae pv. phaseolicola
Source: PLoS One. 2012 Oct 8;7(10):e46815. doi: 10.1371/journal.pone.0046815 (PMC3466206; doi:10.1371/journal.pone.0046815)
Supplement: Table S1 — List of primers used for the amplification of pht genes. (DOC) [file pone.0046815.s002.doc]

Table S1. List of primers used for the amplification of *pht* genes.

| **Amplicon** | **Primer Name** | **Primer sequence (5´→3´)^a^** | **Restriction**  **enzimes** |
| --- | --- | --- | --- |
| ***RT-PCR and Northern blot*** | | | |
| *argK* | L10001 | CTTTGATGGTATGCATGCGGTT |  |
|  | L10002 | GGAAGAACTGGCCAAACATTCG |  |
| ***Construction of plasmids*** | | | |
| *argK* | L100135 | ACATCCGAA**CTGCAG**CCGAGACTAACGAAA | PstI |
|  | L100133 | GTGTTAAAT**CCCGGG**CTCAAAAAGCATACC | SmaI |
| *argK-phtA* | L100135 | ACATCCGAA**CTGCAG**CCGAGACTAACGAAA | PstI |
|  | 1249 | GAACAAG**CCCGGG**ATATAAAAAGGATTCTTCT | SmaI |
| *argK-phtAB* | L100135 | ACATCCGAA**CTGCAG**CCGAGACTAACGAAA | PstI |
|  | L100134 | CAAGTGTC**CCCGGG**CTGTCAAAATCAATAG | SmaI |
| *argK-phtABC* | L100135 | ACATCCGAA**CTGCAG**CCGAGACTAACGAAA | PstI |
|  | 1254 | CCATGGA**CCCGGG**AACTGGTGAGTAGAAGTG | SmaI |
| *phtBC* | L100168 | GTGCTAAA**CCCGGG**ACTCCAGTCGAC | SmaI |
|  | 1254 | CCATGGA**CCCGGG**AACTGGTGAGTAGAAGTG | SmaI |
| *phtC* | L100169 | CATTCGG**CCCGGG**TGAGGTCGAGTAT | SmaI |
|  | 1254 | CCATGGA**CCCGGG**AACTGGTGAGTAGAAGTG | SmaI |
| *phtB* | L100168 | GTGCTAAA**CCCGGG**ACTCCAGTCGAC | SmaI |
|  | L100134 | CAAGTGTC**CCCGGG**CTGTCAAAATCAATAG | SmaI |
| PKA | 1246 | CTTCATG**GGATCC**TCGAGCTCATCAGGGTAA | BamHI |
|  | L100133 | GTGTTAAAT**CCCGGG**CTCAAAAAGCATACC | SmaI |
| PKA-*phtA* | 1246 | CTTCATG**GGATCC**TCGAGCTCATCAGGGTAA | BamHI |
|  | 1249 | GAACAAG**CCCGGG**ATATAAAAAGGATTCTTCT | SmaI |
| PKA-*phtAB* | 1246 | CTTCATG**GGATCC**TCGAGCTCATCAGGGTAA | BamHI |
|  | L100134 | CAAGTGTC**CCCGGG**CTGTCAAAATCAATAG | SmaI |
| PKA-*phtABC* | 1246 | CTTCATG**GGATCC**TCGAGCTCATCAGGGTAA | BamHI |
|  | 1254 | CCATGGA**CCCGGG**AACTGGTGAGTAGAAGTG | SmaI |
| ***DNA Electrophoretic mobility shift*** | | | |
| P_K_ | L100171 | GCTGAATTCAGGGCTTCATAAATTACGG |  |
|  | L100133 | GTGCTAAATCCAGAGCTCAAAAAGCATACC | SmaI |

^a^Restriction sites are indicated in boldface.
